# Comparative Assessment of Gingival Depigmentation Using Scalpel Versus Microneedling With Ascorbic Acid: A Randomized Controlled Trial

**DOI:** 10.7759/cureus.58285

**Published:** 2024-04-15

**Authors:** Swarna Meenakshi P, Subasree S

**Affiliations:** 1 Periodontics, Saveetha Dental College and Hospitals, Saveetha Institute of Medical and Technical Sciences, Saveetha University, Chennai, IND

**Keywords:** ­wound healing, conventional scalpel technique, l-ascorbic acid, gingival depigmentation, microneedling

## Abstract

Background

Gingival pigmentation (GP), characterized by the presence of melanin in the gingival tissues, is a common aesthetic concern in dental practice. While it poses no inherent health risks, the visible discoloration may cause psychological distress for individuals seeking optimal dental aesthetics. Understanding the efficacy of various methods is essential for refining treatment strategies and enhancing patient satisfaction in the realm of gingival depigmentation (GD).

Aim

The objective of the study was to compare the effectiveness of scalpel and microneedling (MN) with ascorbic acid in the treatment of GD.

Materials and methods

Sixteen patients who had a complaint of GP were included in the study, of whom eight were allocated for depigmentation with a scalpel, and the other eight patients were treated with the MN technique with ascorbic acid. Postoperative wound healing scores were evaluated on the first and seventh days, respectively. The intensity of depigmentation was assessed at baseline, in the first month, and at the end of the third month, respectively.

Results

The mean Dummett-Gupta Oral Pigmentation Index (DOPI) score at baseline was 2.65±0.16 and 2.61±0.17 in the surgical and microneedling groups with ascorbic acid, respectively. The mean DOPI score at the end of the third month was 1.67±0.39 and 0.87±0.17 in the scalpel and MN with ascorbic acid groups, respectively. There was a statistically significant difference between the scalpel and MN with ascorbic acid groups at the end of the first and third months, respectively, where MN with ascorbic acid showed aesthetically pleasing outcomes. Patients treated with the scalpel technique showed incomplete healing and ulceration on the first and seventh days after the procedure when compared to the MN technique with ascorbic acid. The healing index scores were statistically significant in the MN with ascorbic acid group.

Conclusion

The MN technique with ascorbic acid is a successful technique for treating GD. It showed aesthetically gratifying outcomes when compared to the conventional surgical technique.

## Introduction

The gingival tissue along the face, lips, and teeth is a significant element in dentofacial aesthetics. In contemporary dentistry, achieving a harmonious dental appearance encompassing both teeth and gingiva is crucial for smile aesthetics [1]. The color of the gingiva plays a pivotal role in overall aesthetics, as visible gingival pigmentation (GP) during speech and smiling can lead to feelings of embarrassment. Patients often find GP aesthetically displeasing, and it can have a psychological impact on them, particularly in individuals with a "gummy smile" or excessive gingival exposure [2]. Beyond addressing biological and functional issues, periodontists are tasked with achieving optimal gingival aesthetics.

Gingival hyperpigmentation is characterized by a darker gingival color that exceeds normal expectations. Pigmentation is a consequence of various physiological processes, encompassing melanin, melanoid, carotene, oxyhemoglobin, reduced hemoglobin, bilirubin, and iron, along with pathological conditions [3]. In particular, melanin pigmentation is the outcome of melanin granules generated by melanoblasts. Furthermore, both active and passive forms of gingival hyperpigmentation are caused by environmental factors, including tobacco use [4]. Age and ethnicity play a role in gingival color, exhibiting no sexual predilection. Various validated procedures for gingival depigmentation (GD) include bur abrasion, scalpel scraping, cryotherapy, electrosurgery, and laser treatments [5]. These methods entail the removal of the epithelial layer along with the underlying connective tissue to promote the regeneration of a new gingival epithelium devoid of melanin. The choice of technique should primarily align with the dentist's clinical expertise and the preferences of the individual patient. Selecting an appropriate method is pivotal for successful GD, considering the varied options available and the importance of personalized care.

The conventional scalpel approach entails the surgical removal of gingival epithelium with a scalpel, allowing the exposed connective tissue to undergo healing through secondary intention [6]. This technique is uncomplicated, cost-effective, and time-efficient, requiring minimal effort. Healing is expedited compared to alternative surgical methods [7]. While surgical gingival stripping offers benefits such as cost-effectiveness and decreased recurrence rates, it is not without its disadvantages. These drawbacks encompass pain, discomfort after the procedure, bleeding during and after surgery, and the need for the application of periodontal dressing [8]. The applicability of this technique is restricted to sites where the gingival phenotype is thin and the interdental papilla is narrow [8].

Microneedling (MN) is a repetitive puncture technique recognized as a form of percutaneous collagen induction therapy [9]. It has gained widespread usage in dermatology for its simplicity, cost-effectiveness, good tolerability, and the dual benefits it provides in both the cosmetic and therapeutic realms. Functioning as a hybrid combining the attributes of hypodermic needling and transdermal patches, MN causes physical trauma by puncturing the stratum corneum layer [10]. Commencing the cascade of wound healing, this procedure generates micro-conduits or small holes with minimal epidermal damage, enabling the swift absorption of topical medications within the stratum corneum layer. As a result, it triggers the release of growth factors, ultimately fostering heightened production of collagen and elastin in the papillary layer [11]. Furthermore, vitamin C has been proven to function as a water-soluble antioxidant and a crucial component in collagen biosynthesis [12]. Vitamin C is pivotal to bone growth, ligaments, teeth, and gums. Beyond its role in collagen formation, ascorbic acid contributes to immunomodulation and the reduction of hyperpigmented spots [13]. This is accomplished by interacting with copper ions at the active site of tyrosinase, leading to the suppression of tyrosinase enzyme activity and, consequently, a reduction in melanin production [14].

Diverse forms of MN have been devised to tackle concerns like scarring, disorders related to pigmentation, and hair loss. Clinical studies have incorporated MN as a component in the treatment of pigmentation disorders, specifically in darker skin types [15]. This includes conditions like vitiligo, melasma, and periorbital hyperpigmentation. Published literature shows that there are limited studies that assess the efficacy of MN in skin pigmentation. Therefore, the objective of the study was to assess the effectiveness of the MN technique in combination with vitamin C in the treatment of GD.

## Materials and methods

The study was conducted at the Department of Periodontics at Saveetha Dental College, Chennai, Tamil Nadu, India. The study protocol received approval from the institutional human ethics committee at Saveetha Dental College, Chennai (ethical clearance number: IHEC/SDC/PERIO-2102/23/301). The trial was registered in the Clinical Trial Registry of India (registration number: REF/2024/03/081656). Before their participation, all individuals provided informed and comprehensive consent. The consent form detailed the treatment regimen, including its benefits, stages, and potential adverse effects. The study enrolled 16 healthy participants with a primary concern related to the treatment of GP. The sample size was estimated using G*Power statistical software (Ver. 3.1 Heinrich-Heine-Universität Düsseldorf, Düsseldorf, Germany), with a power set at 85%.

Inclusion and exclusion criteria

**Figure 1:**
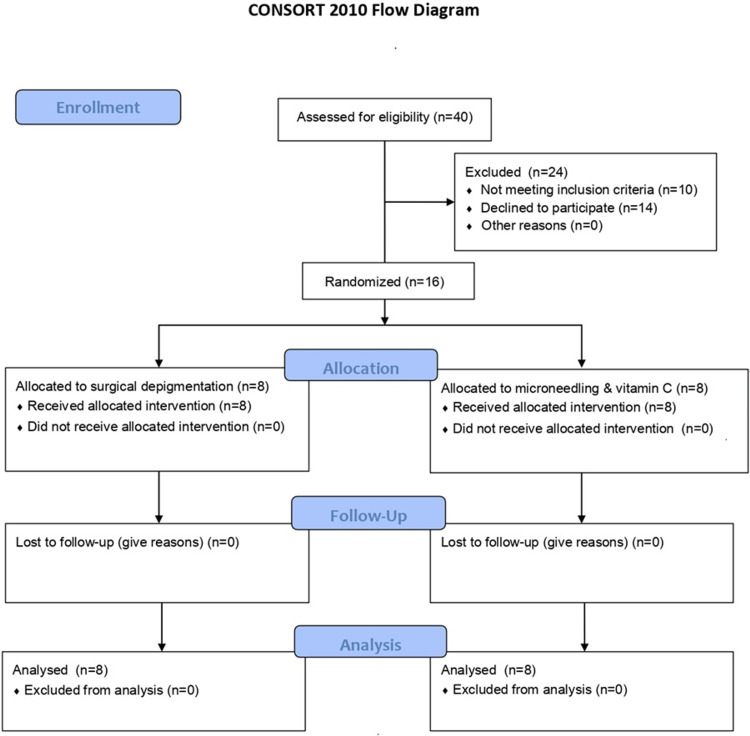
A CONSORT flowchart showcasing the patient selection process The CONSORT flow diagram from the 2010 CONSORT guidelines was selected to decide the inclusion and exclusion criteria for the randomized controlled trial. CONSORT: Consolidated Standards of Reporting Trials

To assess the eligibility of patients for the present study, an initial analysis was conducted, encompassing a thorough review of dental records as well as clinical and radiographic examinations. Sixteen participants meeting specific criteria were selected for the study, including individuals who were aged between 18 and 35 years, demonstrated systemic health, and exhibited physiologic gingival hyperpigmentation within the aesthetic zone. The exclusion of factors potentially triggering an inflammatory response involved screening for systemic disorders, particularly patients with bleeding disorders, individuals undergoing chemotherapy, pregnant women, smokers, gingivitis and periodontitis patients (Figure 1).

Clinical assessments

Intensity of Pigmentation

Patients underwent thorough examination and diagnosis, with the assessment of their pigmentation employing the Dummett-Gupta Oral Pigmentation Index (DOPI) [16], which was designed to gauge the intensity of pigmentation. The assessment criteria for this index were structured as follows: a score of 0 denoted gingiva exhibiting a pink coloration; a score of one indicated a mild light brown hue in the gingiva; a score of two signified a medium brown color or a combination of brown and pink in the gingiva; and a score of three represented a deep brown/blue-black color in the gingiva. Within the mandible, every gingival unit was specifically delineated as comprising an interdental papilla, along with half of the marginal gingiva on both sides. Subsequently, measurements were taken for the associated attached gingiva. The scores were evaluated at baseline, one month later, and three months after the depigmentation procedures.

Wound Healing

The evaluation and scoring of gingival wound healing were performed employing a scale featuring the following criteria: 0 denoting the existence of necrotic gingival tissue, one indicating the existence of a gingival ulcer, two representing incomplete gingival epithelialization, and three signifying complete gingival epithelialization [17]. The cumulative scores for the mandible were employed for analysis, and measurements were recorded on both the first and seventh days postoperatively.

Gingival depigmentation techniques

Scalpel Procedure

**Figure 2:**
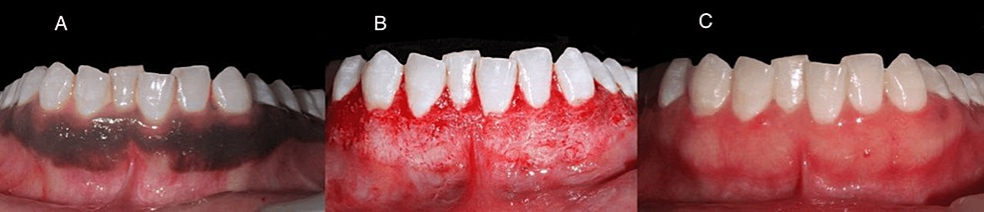
Scalpel depigmentation a) preoperative picture; b) immediate postoperative picture; c) three months after performing the surgical technique

Local anesthesia was administered through infiltration (2% lidocaine with adrenaline 1:200,000) at the surgical site. Employing Bard-Parker (Aspen Surgical, Caledonia, MI) blade number 15, the gingival epithelium in the pigmented area was excised, spanning from the right to left first bicuspids and from the free gingival margin to the mucogingival junction. The blade was oriented roughly parallel to the elongated axis of the teeth, ensuring the comprehensive excision of the epithelium as a unified piece while protecting the underlying bone from exposure. A meticulous assessment of the revealed connective tissue surface was performed, and any residual tissue tags were removed with surgical scissors. Hemorrhage control was managed by applying a pressure pack, and after achieving hemostasis, a periodontal dressing (a non-eugenol zinc oxide dressing) was administered to cover the wound for one week (Figures 2A-2C).

Microneedling Procedure

Prior to the MN procedure, local infiltration anesthesia was administered. In the present study, MN was achieved using a Dermapen (Dr. Pen, Las Vegas, NV) device, and topical ascorbic acid powder (1,000 mg/mL) mixed with saline was applied over the gingiva for 10 minutes.

The device allows for adjustable penetration depth, ranging from 0.2 mm to 3 mm, based on the gingival thickness, and this depth can be set using the device's penetration depth settings. The needle tip comprises 12 to 24 needles arranged in rows, providing six-speed modes that span from 412 cycles/min at the lowest speed to 700 cycles/min at the highest speed. The handpiece empowers healthcare professionals to address areas in any desired orientation. The adjustability of the needle length is facilitated through guides, and the needle tips are designed for disposal, enabling the use of the same handpiece with a new needle tip and guide for diverse patient applications.

**Figure 3:**
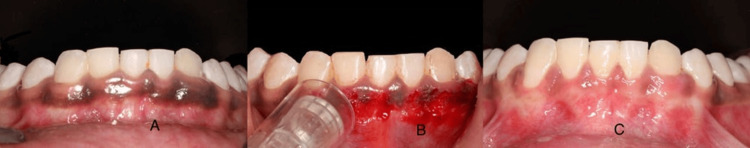
Microneedling technique a) preoperative picture; b) immediate postoperative picture with Dermapen; c) three months after microneedling technique

The assessment of gingival tissue thickness commenced at a location 1.5 mm apical to the marginal gingiva using an endodontic spreader, specifically number 15, fitted with a rubber stopper. The spreader was introduced perpendicularly into the soft tissues until it encountered a firm surface, and the rubber stopper was closely engaged with the gingival surface. Utilizing a digital caliper, the length between the silicone disc and the spreader tip was measured [18]. Adjustments to the microneedle penetration depth were made based on the measured gingival thickness. The Dermapen was positioned perpendicular to the gingival surface, and microneedling was executed horizontally, vertically, and diagonally approximately four to five times across the entire hyperpigmented gingival surface until subtle microbleeding and mild erythema became evident [19]. Each individual underwent a total of three treatment sessions of microneedling, spaced out by 10 days (Figures 3A-3C).

Protocols to Be Followed After the Procedure

Individuals in the scalpel depigmentation cohort were counseled to abstain from mechanical oral hygiene for the initial week post surgery to mitigate potential mechanical harm to the treated area. They were prescribed chlorhexidine mouthwash and an analgesic (tablet Zerodol P, paracetamol 325 mg, and aceclofenac 100mg). In the MN group, patients were directed to avoid mechanical oral hygiene practices specifically for the targeted region on the day of the procedure after each visit. 

Statistical Analysis

The mean and standard deviations of the scores were enumerated. For DOPI, a one-way ANOVA test was used to compare different time spans (baseline, first month, and third month) in the same group, followed by an independent t-test to assess the difference between the groups. To evaluate wound healing scores, an independent t-test was used to calculate the difference between groups, and a paired t-test was utilized to evaluate the difference between time intervals (immediate postoperative and one week) within the same group. Statistical analysis was done using IBM SPSS Statistics for Windows, version 23 (IBM Corp., Armonk, NY). Statistical significance was set at a p-value of less than 0.05.

## Results

The study involved 16 participants who required GD in the mandible. There were no patient dropouts during the course of the study. The mean age of eligible participants was 26.7±5.67 years. Fifty percent were men (n = 8), and 50% were women (n = 8). 

The DOPI score

The mean DOPI scores at baseline were 2.65±0.16 and 2.61±0.17 in the surgical and MN with ascorbic acid groups, respectively. The mean DOPI score at the end of the third month was 1.67±0.39 and 0.87±0.17 in the surgical and MN with ascorbic acid groups, respectively. The outcomes of the current research stated that both the scalpel and MN groups exhibited no statistically significant difference at baseline (p = 0.66), and both the intervened groups showed a statistically significant difference at one month (p <0.03) and the end of the third month (p <0.04) compared to the baseline data (Table 1).

**Table 1:** The mean and standard deviation of DOPI scores between the study groups Group 1: surgical depigmentation; Group 2: microneedling+ascorbic acid A statistically significant difference was seen between Groups 1 and 2 at the end of one month (p = 0.03*) and at the end of the third month (p = 0.04*). Also, a statistically significant difference in values was seen within Group 1 between baseline and one month (p <0.001*) and between baseline and three months (p <0.001*). A statistically significant difference was observed within Group 2 between baseline and one month (p <0.001*) and between baseline and three months (p <0.001*). DOPI: Dummett-Gupta Oral Pigmentation Index

Study groups	DOPI (Group 1)	DOPI (Group 2)	p-value
	Mean±SD	Mean±SD	
Baseline	2.65±0.16	2.61±0.17	0.66
1 month	1.87±0.38	1.07±0.31	0.03*
3 months	1.67±0.39	0.87±0.17	0.04*
P-value (Baseline vs. 1 month)	<0.001*	<0.001*	
(Baseline vs. 3 months)	<0.001*	<0.001*	
(1 month vs. 3 months)	0.26	0.12	

Wound healing score

The healing index exhibited a statistically significant difference between the treated groups (p <0.04) both at baseline and after one week. Patients treated with conventional surgical techniques showed incomplete healing and ulceration on the first and seventh days after the procedure when compared to the MN technique with ascorbic acid. The healing index scores did not show a statistically significant difference within the same group in the surgical technique group at baseline and on the seventh day (Table 2).

**Table 2:** Comparison of the mean and standard deviation of the wound healing scores between the studied groups Group 1: surgical depigmentation; Group 2: microneedling +ascorbic acid A statistically significant difference was seen between Groups 1 and 2 both immediately postoperatively (p = 0.03*) and after one week (p = 0.04*). There was a statistically significant difference in healing index scores between immediate postoperative and one week within Group 2, with a p-value <0.001*. There was no statistically significant difference observed within Group 1 at immediate postoperative and one week (p = 0.32).

Study groups	Healing index (Group 1)	Healing index (Group 2)	p-value
	Mean±SD	Mean±SD	
Immediate postoperative	1.69±0.23	2.29±0.12	0.03*
One week	2.00±0.40	2.90±0.08	0.04*
P-value (Immediate postoperative vs 1 week)	0.32	<0.001*	

## Discussion

Gingival hyperpigmentation is a common concern for many patients seeking cosmetic treatment. Various techniques have been developed for depigmentation of the gingiva, such as scalpel surgery, laser ablation, bur abrasion, and electrocautery. Due to high aesthetic expectations, the optimal strategy should prioritize simplicity and efficiency. The gold standard for addressing GP is surgical intervention, a widely employed procedure. This approach involves excising the complete thickness of the epithelial and papillary connective tissue layers, followed by secondary intended healing of the exposed connective tissue [20]. Surgical depigmentation remains a concern for individuals pursuing improved aesthetics, despite its numerous benefits. Patients commonly report drawbacks such as bleeding, a substantial postoperative wound, pain, and the potential for recurrence. Additionally, in regions with thin gingiva, the surgical technique may lead to the exposure of denuded alveolar bone, further complicating the procedure [21]. As a result, there is a pressing need for alternative, less invasive methods of treating gingival depigmentation. Hence, the MN technique with ascorbic acid was chosen as the supreme technique of the current study.

Recently, MN has emerged as a novel, non-invasive dental technique that can effectively treat GP [22]. The study aimed to compare the efficacy of two distinct treatment modalities, MN with vitamin C and conventional scalpel, in the management of GD. The evaluation was centered around two crucial parameters: the Wound Healing Index (WHI) and the DOPI score. The investigation shed light on the effectiveness of these interventions and provided valuable insights into their respective impacts on wound healing and depigmentation outcomes.

The DOPI score, a pivotal parameter for assessing the degree of depigmentation achieved, also yielded interesting results. The results showed a mean DOPI score of 1.87±0.38 in the scalpel group and 1.07±0.31 in the MN with ascorbic acid group at the end of the first month and 1.67±0.39 in the scalpel group and 0.87 ± 0.17 in the MN with ascorbic acid group at the end of three months, respectively. Both techniques reduced the pigmentation intensity and distribution that were assessed by the DOPI score at one month and three months, respectively, when compared to the baseline scores. In comparison, there was a statistically significant difference between both techniques at the end of the first month and third month, with the MN and ascorbic acid group showing improved DOPI scores. However, the MN with vitamin C group exhibited a more uniform and aesthetically pleasing depigmentation, with a statistically significant difference compared to the scalpel group. This could be attributed to the ability of MN to promote more controlled and even removal of pigmented tissue, resulting in a smoother appearance of the gingiva.

One of the noteworthy findings of the current study was the significant difference observed in the WHI between the MN with vitamin C group and the scalpel group. Healing index scores showed a statistically significant difference between the two groups, with the MN and ascorbic acid group showing complete epithelization on the seventh day when compared to the scalpel technique. The accelerated healing observed in the microneedling group can be attributed to the controlled microtrauma induced by the microneedles, which stimulates collagen synthesis and promotes tissue regeneration. Moreover, the addition of vitamin C likely played a role in mitigating oxidative stress and supporting cellular repair processes [23].

Initially developed for addressing cutaneous hyperpigmentation disorders, the MN technique finds application both independently and in combination with topically applied medications to enhance transdermal drug delivery [24]. Previous research has also evaluated the effectiveness of MN in treating GP. A case report conducted by Mostafa et al. assessed the performance of the MN technique using Dermapen with topical ascorbic acid paste on GD. The study concluded that the MN technique using Dermapen with topical ascorbic acid paste is a successful aesthetic approach for GD [25]. Another study compared the efficiency of vitamin C as a minimally invasive non-surgical technique with the conventional surgical technique of depigmentation. The study concluded that vitamin C is an effective alternative to the conventional surgical technique for the treatment of gingival hyperpigmentation [26]. Moreover, our findings align with the outcomes reported by Shimada et al., wherein the application of topical vitamin C gel on the gingiva demonstrated a melanin pigmentation inhibitory effect [27]. However, El-Mofty et al. conducted a study asserting that intramucosal ascorbic acid injections exhibited superior efficacy compared to topical ascorbic acid gels for treating gingival depigmentation [28].

The current study used Dermapen, which consists of disposable, portable cartridges containing 12 sterilized microneedles. The device ensures a moderate yet constant velocity of automated movements, facilitating more efficient and reliable tissue penetration without inducing deepithelialization [29]. The microscopic wounds induced by MN with Dermapen initiate a regenerative wound repair process. This process involves the release of multiple growth factors crucial for healing, including transforming growth factor, fibroblast growth factor, and platelet-derived growth factor. Ultimately, this cascade of events leads to neocollagenesis, neovascularization, and elastin formation at the junction of the epithelium and connective tissue. The thickening of the stratum spinosum is also observed, collectively contributing to the lightening of hyperpigmented tissues [30]. Microneedling stands out as a cost-effective and less complicated alternative compared to other minimally invasive procedures. Pain is another prominent issue reported by individuals undergoing surgical depigmentation. The discomfort associated with the procedure can significantly affect the patient's quality of life during the recovery phase. Addressing pain management becomes crucial to enhancing patient satisfaction.

It is crucial to acknowledge certain limitations of this study. The sample size and follow-up duration may impact the generalizability of the results. Further, while the study focused on immediate outcomes, long-term follow-ups are essential to assess the sustainability of depigmentation and the persistence of aesthetic results over time. Additionally, patient-specific factors, such as pain perception and acceptance of the treatment modality, should be considered in the broader context of clinical applicability. Consequently, the enduring positive effects of this protocol remain uncertain.

## Conclusions

The outcomes of this study underscore the potential benefits of MN with vitamin C as a viable alternative to conventional scalpel techniques in GD procedures. Beyond its superior wound healing capabilities, MN offers the advantage of precision in depigmentation, reducing the risk of uneven pigmentation commonly associated with scalpel-based procedures. Additionally, the incorporation of vitamin C appears to enhance the overall therapeutic effect, suggesting a synergistic relationship between MN and antioxidant therapy. The MN technique is believed to have a favorable effect on treating GD. Further research must be conducted to understand the limitations of the study and to better understand this new technique. 
